# Expanding the phenotype of biallelic *RNPC3* variants associated with growth hormone deficiency

**DOI:** 10.1002/ajmg.a.61632

**Published:** 2020-05-28

**Authors:** Eline A. Verberne, Sonja Faries, Marcel M. A. M. Mannens, Alex V. Postma, Mieke M. van Haelst

**Affiliations:** ^1^ Department of Clinical Genetics, Amsterdam UMC University of Amsterdam Amsterdam The Netherlands; ^2^ Department of Pediatrics Curaçao Medical Center Willemstad Curacao; ^3^ Department of Medical Biology, Amsterdam UMC University of Amsterdam Amsterdam The Netherlands

**Keywords:** central congenital hypothyroidism, congenital cataract, developmental delay/intellectual deficiency, growth hormone deficiency, minor spliceosome, RNPC3

## Abstract

Pathogenic variants in components of the minor spliceosome have been associated with several human diseases. Recently, it was reported that biallelic *RNPC3* variants lead to severe isolated growth hormone deficiency and pituitary hypoplasia. The *RNPC3* gene codes for the U11/U12‐65K protein, a component of the minor spliceosome. The minor spliceosome plays a role in the splicing of minor (U12‐type) introns, which are present in ~700–800 genes in humans and represent about 0.35% of all introns. Here, we report a second family with biallelic *RNPC3* variants in three siblings with a growth hormone deficiency, central congenital hypothyroidism, congenital cataract, developmental delay/intellectual deficiency and delayed puberty. These cases further confirm the association between biallelic *RNPC3* variants and severe postnatal growth retardation due to growth hormone deficiency. Furthermore, these cases show that the phenotype of this minor spliceosome‐related disease might be broader than previously described.

## 1 INTRODUCTION

Pathogenic variants in components of the minor spliceosome have been associated with several human diseases (Farach et al., 2018; Verma, Akinyi, Norppa, & Frilander, 2018). One of these diseases is isolated growth hormone (GH) deficiency with pituitary hypoplasia, caused by biallelic *RNPC3* variants (Argente et al., 2014). The *RNPC3* gene codes for the U11/U12‐65K protein, a component of the minor spliceosome. The minor spliceosome plays a role in the splicing of precursor mRNA, during which noncoding introns are recognized and removed. Most introns are removed by the major U2‐dependent spliceosome, but a small subset of introns is removed by the minor U12‐dependent spliceosome. These U12‐type introns are present in ~700–800 genes in humans and represent about 0.35% of all introns (Turunen, Niemela, Verma, & Frilander, 2013).

After the publication by Argente et al. (2014), no other cases of biallelic *RNPC3* variants have been reported in the literature, apart from one conference abstract describing two siblings that had isolated GH deficiency and overlapping *RNPC3* variants (Guceva et al., 2015). Here we report novel biallelic *RNPC3* variants in three siblings with GH deficiency, central congenital hypothyroidism, congenital cataract, developmental delay/intellectual deficiency, and delayed puberty.

## 2 CLINICAL REPORT

**FIGURE 1:**
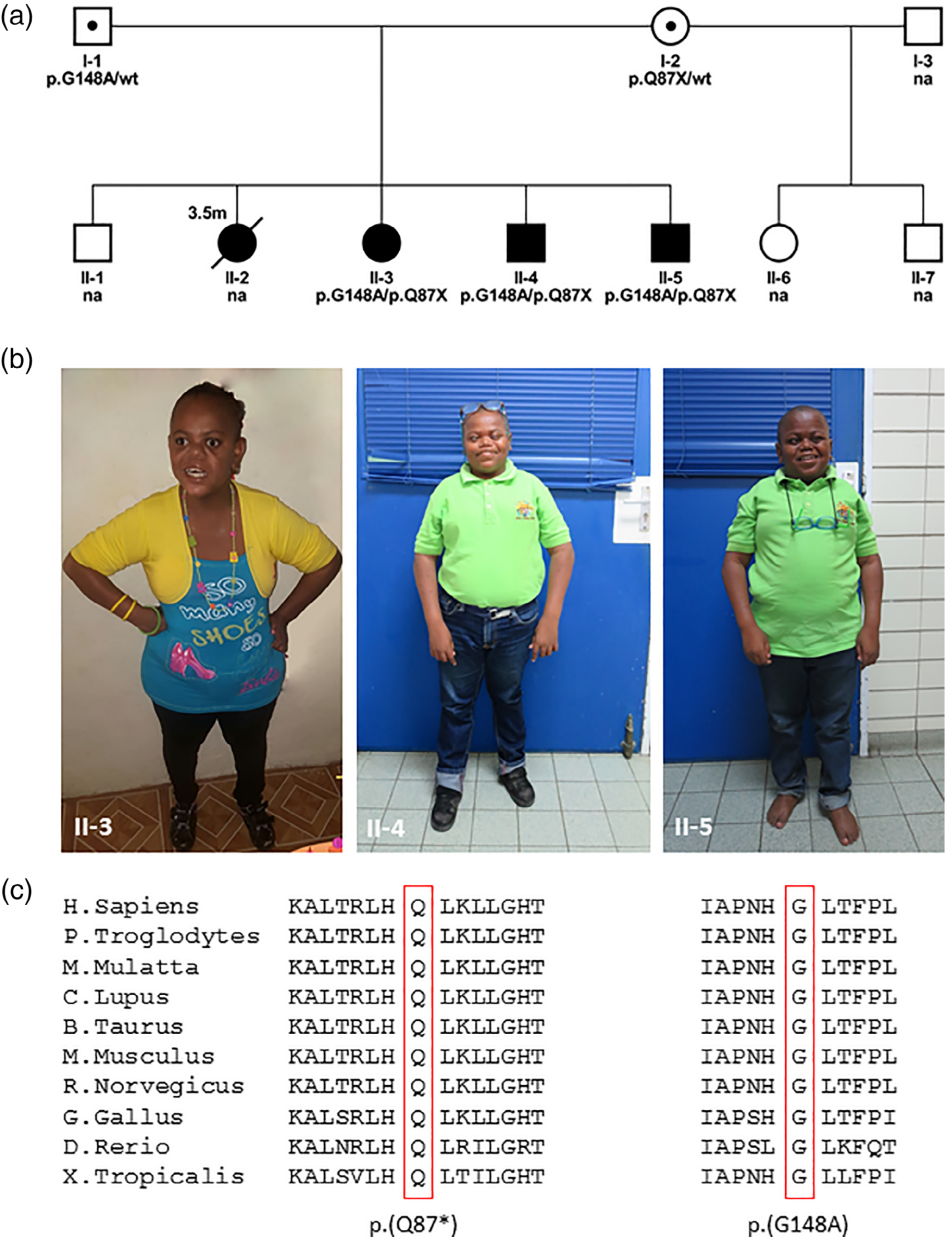
(a) Pedigree showing that the three affected siblings (II‐3, II‐4, and II‐5) are compound heterozygous for the *RNPC3* variants and that both parents are carrier. (b) Patient II‐3, II‐4, and II‐5 at the age of 25, 21, and 17, respectively. Note the short stature, central adiposity, and facial features that are typical of growth hormone deficiency. (c) Amino acid positions of both variants showing complete conservation across vertebrates [Color figure can be viewed at wileyonlinelibrary.com]

Three affected siblings (Figure 1: II‐3, II‐4, and II‐5) were born to healthy, nonconsanguineous Caribbean parents. The mother has two healthy children from a previous relationship. The parents have one healthy older son. Their second child (II‐2) died at the age of 3.5 months, presumably due to aspiration during feeding. This girl was born at term with normal birth weight and had bilateral congenital cataract, hypotonia, hyporeflexia, and absence of sucking reflex, for which she received tube feeding.

The three siblings were born at term after an uncomplicated pregnancy and delivery with normal birth weights. They had congenital cataract, for which they were operated. They all had severe postnatal growth retardation with the height ranging from −6.7 SD to −7.4 SD (Table 1). GH stimulation tests in patient II‐3 and II‐5 showed almost undetectable GH levels (patient II‐4 not tested). Additionally, all three patients had almost undetectable levels of IGF‐1, IGF‐BP3, and prolactin. An X‐ray of the hand was performed in patient II‐3 and II‐4, showing severely delayed bone age (bone age of 6 months at the age of 5 years and 8 months and bone age of 3 months at the age of 2 years and 2 months, respectively). They were diagnosed with central hypothyroidism and received replacement therapy with levothyroxine (patient II‐3 at the age of 7 months and patient II‐4 and II‐5 at the age of 3 months).

**TABLE 1:** Clinical features of previously reported cases and cases reported in this article

	Previously reported					Reported in this article		
Individual	1^a^	2^a^	3^a^	4^b^	5^b^	6 (II‐3)	7 (II‐4)	8 (II‐5)
***RNPC3* variants**	c.1320C > A p.P474T; c.1504C > T p.R502X			c.613C > T p.R205X; c.1420C > A p.P474T		c.259C > T p.(Q87X); c.443G > C p.(G148A)		
**Height (SDS)**	−5.9	−5.0	−6.7	−5	−9	−7.4 (age 7 months)	−6.5 (age 10 months)	−6.7 (age 9 months)
**GH deficiency**	+	+	+	+	+	+	NA	+
**Delayed bone maturation**	+	+	+	+	+	+	+	NA
**Prolactin level**	Low normal	Low normal	Low normal	NR	NR	↓↓	↓↓	↓↓
**Central hypothyroidism**	−	−	−	−	−	+	+	+
**Puberty**	Normal	Delayed	Delayed	NR	NR	Delayed	Delayed	Delayed
**DD and/or ID**	−	−	−	NR	NR	+	+	+
**Brain anomalies**	Hypoplasia of AP	Hypoplasia of AP	Hypoplasia of AP	NR	NR	NA	NA	NA
**Eye problems**	NR	NR	NR	NR	NR	Congenital cataract	Congenital cataract	Congenital cataract

^a^ Argente et al. (2014).

^b^ Guceva et al. (2015).

Abbreviations: ↓↓, almost undetectable; AP, anterior pituitary; DD, developmental delay; GH, growth hormone; ID, intellectual disability; NA, not assessed; NR, not reported; SDS, standard deviation score.

In patient II‐3 GH replacement therapy was started at the age of 1 year. After a short episode of treatment (with a small initial response), almost no effect on growth was noted. The patient did not attend regular follow‐up visits and parents refrained from further use of GH therapy. Because of this noncompliance and since GH therapy is an intensive therapy that requires daily injections and regular monitoring of (adverse) effects, it was decided not to start GH replacement therapy in patient II‐4 and II‐5.

All patients had a developmental delay/intellectual deficiency. At the age of 4, patient II‐3 was able to sit but not stand without support. She had a speech delay, speaking only 2‐word sentences at the age of 5. Neurocognitive examination at this age showed that her development was delayed by 3 years. Patient II‐4 had a motor developmental delay, as he could walk only with support upon examination at the age of 2. During the examination, he was making sounds, although parents indicated that he was able to speak two‐word sentences. At the age of 11, his development was delayed by at least 6 years. Neurocognitive examination in patient II‐5 at the age of 8 showed that his development was delayed by at least 4 years, with an IQ of <42.

Puberty was delayed and laboratory analysis in patient II‐3 and II‐4 was indicative of hypogonadotropic hypogonadism, patient II‐5 was not tested (Table 1).

The patients were evaluated by the visiting clinical geneticist at the ages of 18, 14, and 10‐years‐old, respectively. Height, weight, and head circumference were all well below the third percentile. Apart from short stature, they were noted to have a depressed nasal bridge, short philtrum, and central adiposity (Figure 1).

At the moment, patient II‐3 is 25 years old and she goes to a daycare center on weekdays. Although her IQ has not been formally tested, she appears to have a more severe intellectual deficiency compared to her two younger brothers (patient II‐4 and II‐5). They are now 22 and 18 years old and attend special education.

### 2.1 Genetic testing

Trio whole‐exome sequencing was performed in patient II‐3 in a diagnostic setting as described previously (Houweling et al., 2019). Five variants in three genes were identified: a variant of unknown significance (VUS) in the *HGFAC* gene (c.1228G > C p.[Gly410Arg]), two variants of unknown significance in the *GIF* gene (c.183_186del p.[Met61fs] and c.379G > A p.[Ala127Thr]), a VUS in the *RNPC3* gene (c.443G > C p.[Gly148Ala]) and a pathogenic *RPNC3* variant (c.259C > T p.[Gln87*]). All variants were verified by Sanger sequencing. The variant in the *HGFAC* gene was de novo and did not segregate with the disease, as it was not found in the two affected siblings. Parents were both carriers of one of the variants in the *GIF* gene, however, one of the two affected siblings carried only one *GIF* variant. Thus, these variants were also discarded. The *RPNC3* variants were of particular interest, as biallelic variants in this gene were previously associated with growth hormone deficiency (Argente et al., 2014). Segregation analysis demonstrated that all three affected siblings had the compound heterozygous *RNPC3* variants and that parents were both carriers of one variant (Figure 1). This matches the autosomal recessive mode of inheritance that was expected based upon the pedigree (Figure 1). The pathogenic *RPNC3* variant c.259C > T p.[Gln87*] was present in the Genome Aggregation Database (gnomAD) with an allele frequency of 3.53E‐5, all in the African population and with zero homozygotes, and has a combined annotation‐dependent depletion (CADD) score of 38 (Rentzsch, Witten, Cooper, Shendure, & Kircher, 2019). The c.443G > C p.(Gly148Ala) *RNPC3* variant was not present in gnomAD (https://gnomad.broadinstitute.org/; accessed January 1, 2020) and has a CADD score of 28. Both amino acid positions are completely conserved across vertebrates (Figure 1), indicating that they are likely important for the function of this gene. See also Table S1 for bioinformatic prediction of the *RNPC3* variants in our patients and those previously reported.

No other variants (de novo, homozygous, hemizygous, and/or compound heterozygous) that could be associated with the phenotype were detected. Comparative genomic hybridization (CGH) array analysis was performed in patient II‐3, showing a normal (female) profile.

## 3 DISCUSSION

We here describe three siblings with a combination of growth hormone deficiency, central congenital hypothyroidism, congenital cataract, developmental delay/intellectual deficiency, and delayed puberty with biallelic *RNPC3* variants. These cases further confirm the association between biallelic *RNPC3* variants and severe postnatal growth retardation due to GH deficiency, as previously described (Argente et al., 2014; Guceva et al., 2015). However, our patients show a more extensive phenotype (Table 1).

First of all, the previously described patients had normal levels of other pituitary hormones. This is in contrast to our patients, who had almost undetectable prolactin levels and central congenital hypothyroidism. As brain MRI scans in the patients reported by Argente et al. showed hypoplasia of the anterior pituitary, this could very likely be the cause of the pituitary hormone deficiencies in our patients as well. Unfortunately, this could not be assessed since brain MRI scans are unavailable for our patients. Additionally, our patients had delayed puberty with two of them showing hypogonadotropic hypogonadism upon laboratory analysis. Delayed puberty can be the result of GH deficiency. However, delayed puberty was noticed in two of the three patients initially reported by Argente et al., after treatment with GH for several years, indicating a possible relationship between *RNPC3* variants and impairment of the GnRH axis (Martos‐Moreno et al., 2018).

Secondly, Argente et al. reported normal development, while our patients have a developmental delay and intellectual deficiency. We consider it likely that this could be related to untreated congenital hypothyroidism during the first months of life. When our patients were born there was no newborn screening for congenital hypothyroidism at the Caribbean island, which resulted in a delay in diagnosis and treatment. It is known that thyroid hormone is essential for normal brain development and that untreated congenital hypothyroidism leads to neurocognitive defects (Grosse & Van Vliet, 2011; Kooistra et al., 1994). In addition, patient II‐3 was diagnosed with hypothyroidism only at the age of 7 months and has a more severe intellectual deficiency compared to her younger brothers (patient II‐4 and II‐5), who were diagnosed with hypothyroidism at the age of 3 months. Whole‐exome sequencing revealed no gene defects associated with intellectual deficiency. However, we cannot exclude that the *RNPC3* variants in our patients have contributed to the developmental delay/intellectual deficiency.

Lastly, all three patients were born with congenital cataract. No eye problems were described in the patients reported by Argente et al. and Gucev et al. There are several known environmental causes of congenital cataract. Genetic causes are found in ~10–29% of cases with congenital/infantile cataract (Reis & Semina, 2019). Since congenital cataract was present in all three affected patients and no known environmental causes were identified, a genetic cause is likely. Whole‐exome sequencing revealed no known gene defects associated with congenital cataract. Thus, the congenital cataract in our patients might be a result of the *RNPC3* variants.

The compound heterozygous *RNPC3* variants in the first reported family were functionally studied by Norppa et al. They showed that the nonsense R502X variant resulted in isoform‐specific nonsense‐mediated decay, while the missense P474T variant leads to misfolding and presumably increased decay of the U11/U12‐65K protein. They propose that this causes defective recognition and missplicing of (a subset of) U12‐type introns, leading to impaired pituitary gland development through (yet) unknown mechanisms (Norppa et al., 2018).

We hypothesize that biallelic pathogenic variants in *RNPC3* can lead to a spectrum of disease, with patients on the severe end having not only GH deficiency, but deficiency of other anterior pituitary hormones as well. We further hypothesize that, on the severe end of the spectrum, *RNPC3* variants could lead to defective splicing of genes that play a role in the development of the eyes and possibly also the brain. In line with this, it has been found that pathogenic variants in *RNU4ATAC*, another gene that encodes a component of the minor spliceosome, are associated with three distinct clinical conditions (microcephalic osteodysplastic primordial dwarfism type 1 [MOPD1], Roifman syndrome and Lowry Wood syndrome) that differ in severity but have overlapping features (Farach et al., 2018). There is some evidence for genotype–phenotype associations in these *RNU4ATAC* ‐associated disorders, which could partially explain the clinical differences (Shelihan et al., 2018). Similarly, there might be a genotype–phenotype association in patients with biallelic variants in *RNPC3*, as the variants reported in our family differ from those reported before. However, not enough patients have yet been reported to evaluate if such an association truly exists. Additionally, modifier genes could (partly) explain the phenotypic variation between patients with *RNPC3* variants.

Of further interest, there have been reports of patients with MOPD1 that had bilateral cataract, which was a feature in our patients as well (Kilic et al., 2015; Kroigard et al., 2016).

In conclusion, we show that the phenotype associated with biallelic *RNPC3* variants is broader than previously described. The exact mechanisms through which pathogenic *RNPC3* variants cause different phenotypes still remain to be elucidated.

## DISCLOSURE AND CONSENT

The authors declare that they have no conflict of interest. Written informed consent for publication was obtained from the mother of the three patients.

## AUTHOR CONTRIBUTION

Eline Verberne contributed to the study design, interpretation of the data and manuscript writing, under the supervision of Marcel Mannens and Mieke van Haelst. Sonja Faries was involved in the collection and interpretation of the clinical data. Alex Postma contributed to the analysis and interpretation of the genetic data. All authors were involved in revising the manuscript and approved the final version.

## Supporting information


**Table S1**

## Data Availability

Data sharing is not applicable to this article as no new data were created or analyzed in this study.
