# Supplementary material for: Expanding the phenotype of biallelic RNPC3 variants associated with growth hormone deficiency
Source: Am J Med Genet A. 2020 May 28;182(8):1952–6. doi: 10.1002/ajmg.a.61632 (PMC7496482; doi:10.1002/ajmg.a.61632)
Supplement: Supplementary file 1 — Table S1 [file AJMG-182-1952-s001.docx]

| **Source** | **Chr** | **Pos (hg38)** | **Ref** | **Alt** | **Consequence** | **oAA** | **nAA** | **protPos** | **SIFT** | **PolyPhen** | **mamPhCons** | **verPhCons** | **CADD score** |
| --- | --- | --- | --- | --- | --- | --- | --- | --- | --- | --- | --- | --- | --- |
| Reported in this paper | 1 | 103533757 | C | T | STOP_GAINED | Q | * | 87 | NA | NA | 1 | 1 | 38 |
| Reported in this paper | 1 | 103534857 | G | C | NON_SYNONYMOUS | G | A | 148 | deleterious | probably_damaging | 1 | 1 | 27.6 |
| Previously reported^1^ | 1 | 103536183 | C | T | STOP_GAINED | R | * | 205 | NA | NA | 1 | 0.999 | 36 |
| Previously reported^1,2^ | 1 | 103550999 | C | A | NON_SYNONYMOUS | P | T | 474 | deleterious | probably_damaging | 1 | 1 | 26.0 |
| Previously reported^2^ | 1 | 103551730 | C | T | STOP_GAINED | R | * | 502 | NA | NA | 1 | 1 | 38 |

^1^Guceva, Z., Polenakovicb, M., Tasica, V., LeBoucc, Y., Klammtd, J., Pfaeffled, J., & Filipovskae, A. (2015). Severe isolated growth hormone deficiency and myopathy in two brothers with RNPC3 mutation. Horm Res Paediatr, 84 (Suppl 1), 447.

^2^Argente, J., Flores, R., Gutierrez-Arumi, A., Verma, B., Martos-Moreno, G. A., Cusco, I., . . . Perez-Jurado, L. A. (2014). Defective minor spliceosome mRNA processing results in isolated familial growth hormone deficiency. EMBO Mol Med, 6(3), 299-306. doi:10.1002/emmm.201303573
